# Targeting Polyprotein to Design Potential Multiepitope Vaccine against *Omsk Hemorrhagic Fever Virus* (OHFV) by Evaluating Allergenicity, Antigenicity, and Toxicity Using Immunoinformatic Approaches

**DOI:** 10.3390/biology13090738

**Published:** 2024-09-20

**Authors:** Abdullah M. Alnuqaydan, Alaa Abdulaziz Eisa

**Affiliations:** 1 Department of Basic Health Sciences, College of Applied Medical Sciences, Qassim University, Buraidah 51452, Saudi Arabia; 2 Department of Medical Laboratories Technology, College of Applied Medical Sciences, Taibah University, Meddina 30002, Saudi Arabia; aeisa@taibahu.edu.sa

**Keywords:** toxicity, virus vaccine, fever, antiviral therapy, immunoinformatic

## Abstract

**Simple Summary:**

The current study aimed to design a multiepitope subunit vaccine against the Omsk Hemorrhagic Fever Virus (OHFV). The predicted potent antigenic, non-allergenic, and nontoxic cytotoxic T-lymphocyte (CTL), helper T-lymphocyte (HTL), and linear B-lymphocyte (LBL) epitopes were chosen considering several vaccine parameters. After screening, eight (8) CTL, five (5) HTL, and six (6) B cell epitopes were joined. The designed vaccine construct was predicted to generate robust primary and secondary immune responses. In addition, the vaccine construct was blindly docked to the TLR4 immune receptor and subjected to conformational dynamics simulation analysis to interpret its intermolecular binding affinity and time-dependent dynamic behavior.

**Abstract:**

Omsk Hemorrhagic Fever Virus (OHFV) is an RNA virus with a single-stranded, positive-sense genome. It is classified under the Flaviviridae family. The genome of this virus is 98% similar to the Alkhurma hemorrhagic fever virus (AHFV), which belongs to the same family. Cases of the virus have been reported in various regions of Saudi Arabia. Both OHFV and AHFV have similarities in pathogenic polyprotein targets. No effective and licensed vaccines are available to manage OHFV infections. Therefore, an effective and safe vaccine is required that can activate protective immunity against OHFV. The current study aimed to design a multiepitope subunit vaccine against the OHFV utilizing several immunoinformatic tools. The polyprotein of OHFV was selected and potent antigenic, non-allergenic, and nontoxic cytotoxic T-lymphocyte (CTL), helper T-lymphocyte (HTL), and linear B-lymphocyte (LBL) epitopes were chosen. After screening, eight (8) CTL, five (5) HTL, and six (6) B cell epitopes were joined with each other using different linkers. Adjuvant human beta defensin-2 was also linked to the epitopes to increase vaccine antigenic and immunogenic efficiency. The designed vaccine was docked with Toll-like receptor 4 (TLR4) as it activates and induces primary and secondary immune responses against OHFV. Codon optimization was carried out, which resulted in a CAI value of 0.99 and 53.4% GC contents. In addition, the construct was blindly docked to the TLR4 immune receptor and subjected to conformational dynamics simulation analysis to interpret the intricate affinity and comprehend the time-dependent behavior. Moreover, it was predicted that immune responses to the developed vaccine construct reported formation of strong humoral and cellular immune cells. Therefore, the proposed vaccine may be considered in experimental assays to combat OHFV infections. Laboratory experiments for the above predictions are essential in order to evaluate the effectiveness, safety, and protective properties of the subject in question.

## 1. Introduction

Omsk Hemorrhagic Fever (OHF) is an infectious disease caused by *Omsk Hemorrhagic Fever Virus* (OHFV), a single-stranded, positive-sense RNA virus [1]. The OHFV belongs to the family Flaviviridae and is placed in the Orthoflavivirus genus [2]. The incubation period of OHFV is 6–14 days [3]. During the incubation time, infected individuals may experience non-specific flu-like symptoms before the primary symptoms. Primary symptoms include cough, headache, chills, nausea, muscle pain, subconjunctival hemorrhage, gastrointestinal symptoms, skin hemorrhages with fever, uterine, gingival, and nasal bleeding. Moreover, patients may experience upper body petechial rashes and hyperesthesia (severe skin sensitivity) [1,4]. In the secondary phase, along with the primary symptoms, the patient developed encephalitic symptoms, including meningitis and headache, which occur in 30–50% of patients [5]. The patient may experience long-term consequences like the loss of hearing, loss of hair, and mental health issues, along with neurological dysfunction in certain cases [6]. Very few patients advance to hemorrhagic complications. Thus, the OHFV case fatality rate is around 0.5–3%, which is much lower than other flaviviruses [4].

In a comparative analysis, in 2014, Madani and colleagues characterized the complete genome sequence of an Alkhurma hemorrhagic fever virus (AHFV) strain obtained from a patient in Najran. They aligned it with sequences of the OHFV and found that it shows 98% identity [7]. OHFV is found in *Dermacentor reticulatus*, formerly known as *D. pictus*, found in the Siberian steppes and forests, while in the southern and western Siberian steppes, OHFV is found in *D. marginatus* [8,9]. They obtain their food from humans, small mammals, domestic animals, and ungulates and act as parasites on numerous birds and mammals in Russia [3]. OHFV is transmitted to humans through the bite of these ticks or via nutritive or respiratory pathways when a person encounters an infected animal. Person-to-person transmission has not been reported yet [10]. The *Ixodes persulcatus* tick also plays a role in the life cycle of OHFV. Moreover, OHFV was identified and isolated from various mosquitos like *Ochlerotatus excrucians*, *Oligoryzomys flavescens,* and *Coquilletidia richiardii*, but the data did not show any role in OHFV ecology [11]. Muskrats (*Ondathra zibethicus*), a common mammal in Europe, North America, Russia, the Balkans, and Kazakhstan, have a high viral load of OHFV and can transmit the virus to healthy mammals [3].

Between the years 1945 and 1958, the first outbreak occurred in Russia, and more than 1500 suspected and 927 confirmed cases of OHF were reported. Over 300 OHF cases were confirmed by 1998 in the Omsk, Tyumen, Novosibirsk, and Kurgan regions [12]. In research conducted between 1970 and 1975, 18% of the 577 patients with fever of unknown origin (FUO) had been exposed to OHFV. The findings indicated that, irrespective of gender, people with a mean age between 20 and 40 were most affected, along with 30% of children under the age of 15 [13]. No data about the recent OHF outbreaks in Russia, or any other place, are available in any journals due to courses being mild and undetected [4].

OHFV is polygonal or spherical, having a 40 nm diameter with a 25 nm nucleolus. The nucleocapsid of OHFV consists of a single-stranded, positive-sense RNA genome enfolded in capsid protein and enclosed in a lipid bilayer derived from the host cell. The entire genome of the OHFV comprises 10,787 nucleotides, with a 10,242-nucleotide-long open reading frame (ORF) region [4,14]. The open reading frame is encoded by a polyprotein 3414 amino acids long that is cleaved at specific sites by viral and cellular proteases after or during translation. The polyprotein makes three structural and seven non-structural proteins. C, prM, and E are structural, while NS1, NS2A, NS2B, NS3, NS4A, NS4B, and NS5 are non-structural proteins [15].

OHFV is a tick-borne flavivirus that can seriously sicken people. Because OHFV’s polyprotein is processed into several structural and non-structural proteins necessary for the virus’s replication and virulence, it is largely responsible for the virus’s pathogenicity. OHFV causes hemorrhagic signs, headaches, feverish episodes that come on suddenly, and muscle aches in people. Severe instances may result in neurological issues. It is essential to comprehend the function of the polyprotein and its processed components to create antiviral treatments and possible OHFV vaccinations [16].

According to research, the OHFV has a strong attraction for vascular and hemopoietic tissues (vascular and hematopoietic tropism, respectively). Researchers have also investigated OHFV pathogenesis in non-human primates and in vivo mice models. It was identified that the infection of OHFV in BALB/c mice resulted in serious cerebellar abnormalities and mild meningoencephalitis, which are symptoms that have been previously reported in patients infected with OHFV [10,17]. Moreover, the highest level of viral load was observed in the brain hemispheres and cerebellum, followed by lungs, spleen, blood, feces, liver, and kidneys [18]. One trait that distinguishes the pathogenic processes in mice is the persistence of the virus in infected subcutaneous tissues and internal organs. When infections are severe, the OHFV enters the brain through the blood–brain barrier, leading to the activation of the central nervous system (CNS) [10]. There is no vaccine or medication specifically designed yet for the prevention and cure of OHF, although the manifestations of disease can be reduced by treating the symptoms [19]. During the 1991 outbreak, vaccinations against tick-borne encephalitis (TBE) were utilized as a prophylactic strategy to treat OHFV due to antigenic similarities. However, no clinical evidence demonstrated the effectiveness of vaccine against the OHFV [3,10].

The present study aimed to designed an in silico multiepitope vaccine construct with ability to induce immunity against OHFV. The multiepitope vaccine is predicted to be highly effective, specific, and thermodynamically stable. Different immunoinformatic methods were utilized for the selection of highly antigenic, non-allergenic, non-toxic, and non-overlapping epitopes to design a vaccine construct. The predicted T cell epitopes derived from B cells in those who were chosen were then used in simulation and docking assays to figure out the construct’s affinity for the TLR4 receptor as a test case [20] and to search for conformational changes in both the construct and the receptor that could influence the construct binding. It is anticipated that the chimeric construct will be very successful in managing infections brought on by this pathogen. Vaccinologists may find it advantageous to assess the construct’s immunoprotected capabilities in animal models. The primary goal of the study, given the computational nature of the research, is to give experimentalists a theoretical vaccination model that they may use to verify the intended vaccine’s immune protective effectiveness against the specified pathogen in vivo. In recent times, there has been an increased focus on reverse vaccinology (RV) as a method for identifying vaccine proteins against various infections [21]. The licensed Bexsero vaccine was developed as a result of the RV technique, which was initially used to target the bacterial disease Meningococcus B (MenB) [22]. RV played a crucial role in determining the antigen that demonstrated the broadest bactericidal effect and in terminating the lengthy procedure of creating the MenB vaccination. RV has also been used to treat a wide range of other bacterial infections, including as group A streptococcus, streptococcus pneumonia, antibiotic-resistant Staphylococcus aureus, and chlamydia. Additionally, experimental research has demonstrated the effectiveness of peptide- or subunit-based vaccinations that were first discovered using an RV technique [23,24]. Eight (8) CTL, five (5) HTL, and six (6) B Cell epitopes were found to be good candidates for vaccine development in this work using an RV method to screen potential vaccine proteins against the OHFV. This work will speed up development of a vaccine against this virus by providing opportunities for experimentalists to investigate the immune protection efficiency of the selected epitopes in animal models.

## 2. Material and Methods

### 2.1. Retrieval of Sequence

The amino acid sequence of polyprotein (Uniprot ID: Q6JJM0), (GenBank ID AY438626.1) of OHFV was retrieved from UniProtKB database in Fasta format. The retrieved sequence was then processed for epitope prediction to design multiepitope vaccine against OHFV.

### 2.2. Major Histocompatibility Complex-I (MHC-I) Epitopes Prediction

NetCTL1.2 server (Lyngby, Denmark)was utilized to predict CTL epitopes related to MHC-I at 0.75 thresholds. The server determined binding affinity along with the COMB score of the MHC-I epitope. The server predicted CTL epitopes on the basis of Transporter-Associated Antigen Processing TAP and C-terminal cleavage scores. For the determination of C-terminal cleavage scores and weight matrix to computed TAP score, an artificial neural network was employed by the server [25].

### 2.3. Epitope Prediction for Major Histocompatibility Complex-II (MHC-II)

Immune Epitope Database (IEDB), a web server, was used to predict MHC-II epitopes (http://www.iedb.org/) (accessed on 25 March 2024) [26]. The server predicts the binding of MHC-II epitopes with seven (7) human reference HLAs, specifically including HLA-DRB3*01:01, HLA-DRB1*07:01, HLA-DRB4*01:01, HLA-DRB5*01:01, HLA-DRB1*03:01, HLA-DRB3*02:02, and HLA-DRB1*15:0, which are based on IC_50_ scores assigned by server to each MHC-II epitope. There is an inverse relation between IC_50_ score and binding affinity: the lower the IC_50_ score, the higher the binding affinity of MHC-II epitopes. Contrarily, the binding affinity of the epitopes will be higher if the IC_50_ score is lower. There is also an inverse relation between percentile rank and MHC-II epitopes [27,28]. Lower percentile rank and non-overlap epitopes were further processed for vaccine designing.

### 2.4. B Cell Epitope Prediction

B-lymphocyte produced specific antibodies and memory cells for neutralization and long-term immunological protection against specific viral invaders [29]. B cells in the body of host are activated when epitopes are attached to the B cell receptor [30]. BCpred is an online server (http://ailab-projects2.ist.psu.edu/bcpred) (accessed on 26 March 2024) that uses anticipated B cell epitopes and SVM techniques. High-scoring B cell epitopes were selected for in silico vaccine development [31].

### 2.5. Epitopes Evaluation for Vaccine Development

Numerous parameters including toxicity, allergenicity, and antigenicity were considered for selecting B cell, CTL and HTL epitopes to construct multiepitope vaccine. VaxiJen online server was employed for antigenicity prediction using 0.4 threshold as default value [32]. Algepred was utilized for allergenicity with −0.4 threshold value [33]. All the antigenic and non-allergenic epitopes were uploaded to an online server ToxinPred (http://crdd.osdd.net/raghava/toxinpred/) (accessed on 26 March 2024). To check toxicity of each epitope [34]. Non-overlapping, highly antigenic, non-allergenic, and non-toxic epitopes were selected for vaccine construction.

### 2.6. Multiepitope Vaccine Construct

The epitopes that matched all the criteria were subsequently selected for vaccine design. The linkers AAY, GPGPG, and KK were specifically used to combine CTL, HTL, and B cell epitopes, respectively. In addition, an adjuvant called human beta-defensin-2 was attached to the N-terminus of the vaccine construct using EAAAK linker to improve the stability and potential of vaccine [27,28]. The vaccine construct was subsequently assessed for antigenicity and allergenicity using VaxiJen and Algepred servers.

### 2.7. Physiochemical Properties Prediction

The Physiochemical characteristics of the designed vaccine were determined using the ProtParam server (http://web.expasy.org/protparam/) (accessed on 28 March 2024) [35]. This analysis evaluated the Aliphatic and instability index, GRAVY, theoretical PI, in vitro and in vivo half-life, and molecular weight of the vaccine design [36].

### 2.8. Prediction of Secondary and Tertiary Structure of the Vaccine Construct

The PSIPREDV3.3 server, available at http://bioinf.cs.ucl.ac.uk/psipred/ (accessed on 29 March 2024), was used to forecast the secondary structure of the vaccine as described by McGuffin, Bryson, and Jones (2000) [37]. The tertiary structure was determined using the internet server Robetta (http://robetta.bakerlab.org) (accessed on 30 March 2024) [38], which utilizes comparative modeling techniques based on PSI-BLAST, 3D-Jury, FFAS03, and BLAST to identify the template structure for the vaccine peptide sequence. In some instances when the template for the given sequence is unavailable, the server employs the de novo Rosetta insertion fragment approach.

### 2.9. Validation of Tertiary Structure

The validation of tertiary structure of constructed vaccine was carried out through ProSA-web (https://prosa.services.came.sbg.ac.at/prosa.php) (accessed on 4 April 2024) [39], PROCHECK [40], ERRAT [41], and ProQ (protein quality predictor) servers [42]. Ramachandran Plot analysis, non-bonded linkages, and Z-score was computed by the servers and verified the structure of vaccine construct [43].

### 2.10. Molecular Docking of TLR4 and Vaccine Construct

Cluspro docking server (https://cluspro.bu.edu/login.php) (accessed on 10 April 2024) was utilized for determining the interface between TLR4 and vaccine construct [44]. The server is based on the nature of protein and six energy functions and provided ten (10) docking solutions in approximately 4h. Each cluster is immensely crowded with lowest energy parameter, which explains ranges of choices including numerous energy parameters, resulting in extra limited files and result analysis. Moreover, PDBsum was utilized for graphical representation of different residues interference between TLR4 and vaccine construct [25].

### 2.11. Codon Optimization of Vaccine Construct and In Silico Cloning

The process of codon optimization was carried out by utilizing the Jcat (version 1.0) software, resulting in the acquisition of an ideal DNA sequence. Jcat assessed the Codon Adaptation Index (CAI) and GC levels. The Prokaryotic Ribosome interface site, cleavage site for restriction enzymes, and Rho-independent termination of transcription were chosen to ensure the targeted expression of vaccine construct in specified vector. The DNA sequence was flanked by XhoI and NdeI restriction sites at the 5′ and 3′ ends, respectively. The DNA sequence of the vaccine was subsequently inserted into the pET28a (+) cloning vector using the SnapeGene (version 1.1.3) program [45].

### 2.12. In-Silco Immune Responses Analysis

In silico immune simulations were performed by the online server C-ImmSim (http://150.146.2.1/C-IMMSIM/index.php) (accessed on 15 April 2024), which determined the immunogenic response of the designed vaccine in actual life [46]. This server operates as an agent-based dynamic simulator, forecasting interactions among immune systems and epitopes prediction through machine learning procedures and PSSM, which is the specific scoring matrix. Each injection composed of 1000 vaccine units provided to our immune simulation at a gap of four weeks. The other parameters of the server were kept at default setting. After the administration of vaccine sequence antibodies, interferon and cytokines were evaluated by server [47].

### 2.13. Molecular Dynamics Simulations

The top docked complex was exposed to MD simulation for 100 ns following molecular docking to display its time-dependent behavior [48]. The tLeap program of AMBER16 was used for system preparation, while Sander was used for preprocessing. According to [49], the system was solvated in a 12 Å TIP3P water box [50]. In the preprocessing stage, the water box and complex hydrogen atoms were reduced for 500 and 1000 cycles, respectively. In addition, the system was reduced for 1000 steps with constraints of 5 kcal/mol/Å^2^ on alpha carbon atoms. Ultimately, 300 rounds of system reduction were accomplished with 100 kcal/mol constraints on non-heavy elements. The temperature was scaled to 300 K for 20 ps using the Langevin dynamics [51] and the SHAKE algorithm was used into the complex for 100 nanoseconds while applying a 5 kcal/mol/Å^2^ constraint to the C-alpha atoms [52]. Using isotropic position scaling and the ensemble of NPT, the system’s pressure was sustained for 50 ps. In the end, the NVT ensemble and the Berendsen algorithm were used to produce 100 ns [53]. The CPPTRAJ module of AMBER was utilized to calculate the RMSD and RMSF analysis [48].

## 3. Results

### 3.1. Sequence Retrieval of Polyprotein

Polyprotein from the virus was retrieved from Uniprot data bank in Fasta format. CTL, HTL, and linear B cell epitopes were predicted using online servers for the construction of the multiepitope.

### 3.2. MHC-I Epitopes Prediction and Evaluation

The CTL epitopes of the OHFV polyprotein were predicted by using the NetCTL1.2 server. CTL epitopes having a high scoring value means a high binding affinity with the MHC-I receptor. A CTL with a high binding affinity was selected the for vaccine designing. Moreover, the selected epitopes were evaluated for allergenicity, antigenicity, and toxicity. Eight (8) CTL epitopes gratified the desired parameters and were processed for vaccine designing. The detail of CTL epitopes are given in Table 1.

### 3.3. MHC-II Epitope Prediction and Evaluation

The IEDB online server was employed for prediction of HTL epitopes. These epitopes are linked with MHC-II peptides and trigger Helper-T-lymphocytes (HTL). The server uses a set of seven reference human leukocytes (HLAs), which are HLA-DRB3*01:01, HLA-DRB4*01:01, HLA-DRB1*03:01, HLA-DRB1*15:01, HLA-DRB1*07:01, HLA-DRB5*01:01, and HLA-DRB3*02:02, to predict HTL epitopes. The predicted epitopes were processed for allergenicity, antigenicity, and toxicity. Subsequently, five (5) HTL epitopes with low percentile rank, non-overlapping features, and demonstrated antigenicity, non-allergenicity, and non-toxicity were shortlisted for vaccine design (as indicated in Table 2).

### 3.4. B Cell Epitopes Prediction and Assessment

The BCpreds server was employed to forecast the epitopes of linear B-lymphocytes (LBL) inside the OHFV polyprotein. Each epitope was assigned a binding score, which indicated its level of affinity to the targeted peptide. Epitopes that had higher binding scores were regarded as being highly antigenic, devoid of toxicity, and non-allergenic. Given these factors, we have chosen six (6) epitopes for more research. Crucially, we have verified that the selected epitopes do not intersect with one another, as shown in Table 3.

### 3.5. Construction of Multiepitope Vaccine and Assessment

**Figure 1:**
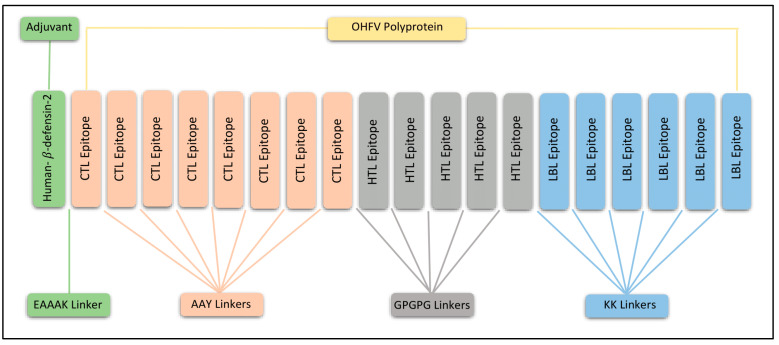
Graphical representation of vaccine construct along with adjuvant, selected CTL, HTL, LBL epitopes, and linkers (EAAAK, AAY, GPGPG, KK).

We thoroughly assessed selected epitopes using many criteria and integrated them into a specifically built multiepitope vaccination structure. Distinct linkers were used to connect CTL, HTL, and B cell epitopes: AAY linkers for CTL epitopes, GPGPG linkers for HTL epitopes, and KK linkers for B Cell epitopes. In addition, we strategically connected an adjuvant called Human-beta-defensin-2 to the N-terminus using an EAAAK linker. This connection improves the effectiveness of the vaccine, as seen in Figure 1. The vaccine build consists of 375 amino acids. The produced vaccine had a notable antigenicity score of 0.74 and a non-allergenic score of −0.94, showing a high level of both antigenicity and safety. In addition, we evaluated the physicochemical characteristics, thereby verifying the durability of the developed vaccine. Table 4 contains comprehensive information on the qualities.

### 3.6. Prediction of Secondary and Tertiary Structure

**Figure 2:**
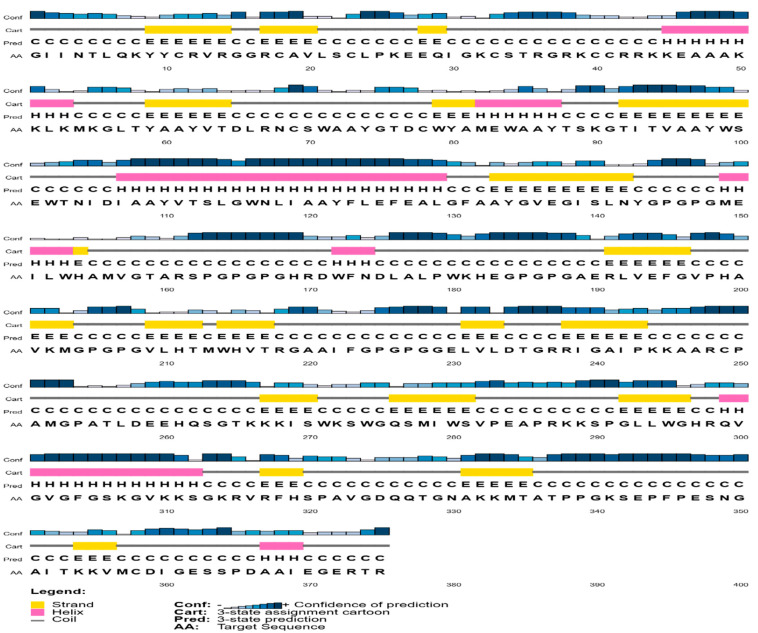
The vaccine construct’s secondary structure is depicted with the expanded coil represented by black lines, the beta strand by a yellow bar, and the alpha helix by a pink bar.

**Figure 3:**
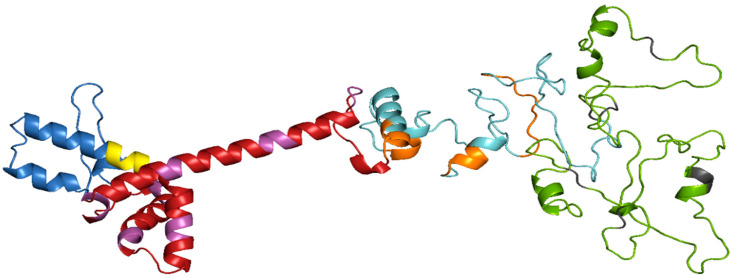
Tertiary structure representation of vaccine. The adjuvant is shown in blue color, EAAAK linker in yellow color, CTL epitopes in red color, AAY linker in pink color, HTL epitopes in cyan color, GPGPG linker in orange color, B Cell epitopes in green, and KK linker in gray color.

**Figure 4:**
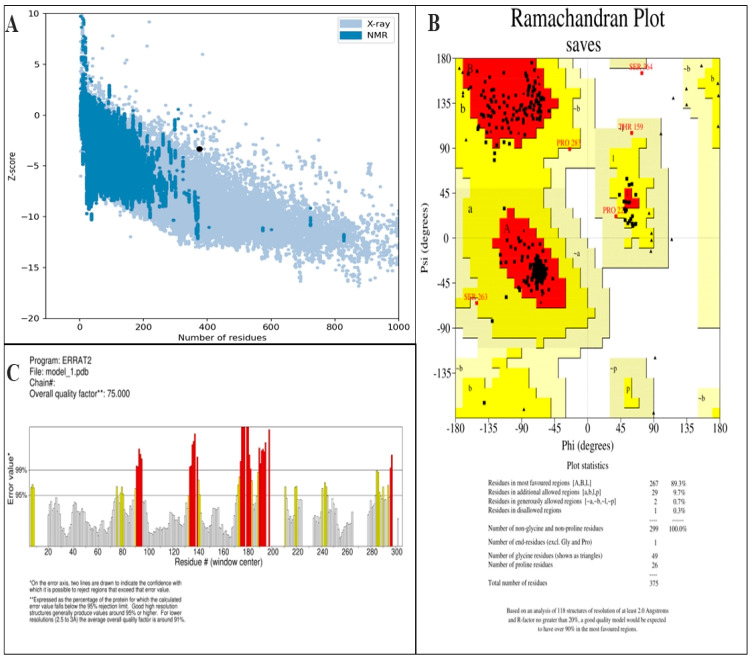
Tertiary structure validation of vaccine construct. (**A**) represents ProSA-web analysis, (**B**) represents Ramachandran plot, and (**C**) represents ERRAT analysis. The Ramachandran plot coloring is detailed at https://www.ncbi.nlm.nih.gov/pmc/articles/PMC5734310/ accessed on 20 April 2024.

The amino acid sequence of the vaccine was submitted to the PSIPREDV3.3 service in order to forecast its secondary structure. The vaccine design exhibited 24.8% beta strand, 16.8% alpha helix, and 58.4% extended coils. The specific breakdown of secondary structures may be seen in Figure 2. In addition, the Robetta server was used to estimate the tertiary structure of the planned vaccine. The Robetta server has generated predictions for the top five 3D models of the vaccine build. The optimal model was chosen, as seen in Figure 3, after assessment on many servers such as ProSA-web, Ramachandran plot, Protein Quality Predictor (ProQ), and ERRAT. The Z-score determined by ProSA-web was −3.34, indicating that the input structure score is higher than that of a natural protein of the same size, as seen in Figure 4A. The PROCHECK server used a projected Ramachandran plot to calculate the percentages of residues in different regions. The most favored area contains 89.3% of the residues, the extra allowed region contains 9.7% of the residues, the generously permitted region contains 0.7% of the residues, and the prohibited region contains 0.3% of the residues, as shown in Figure 4B. The ProQ model accurately predicted the LGscore and MaxSub value to be 11.228 and −1.026, respectively, indicating that the model is of exceptional quality. In addition, the ERRAT server assessed the 3D model and determined an overall quality factor of 75%, as seen in Figure 4C.

### 3.7. Investigation of the Interaction between the Vaccine Design and TLR4

**Figure 5:**
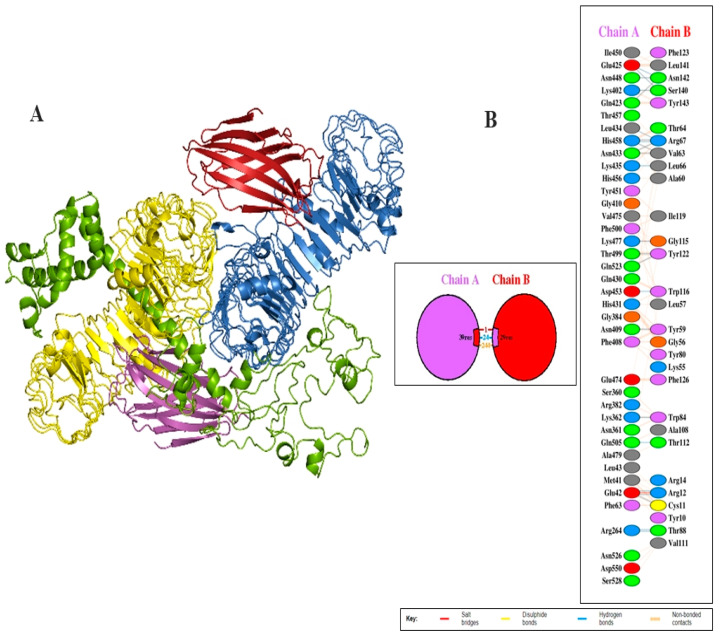
Docked complex system of vaccine construct with TLR4 (**A**) representation of docking complex (Chain A in blue color, Chain B in yellow color, Chain C in red color, and Chain D in magenta color) while split pea color represents vaccine molecule. (**B**) Graphical illustration of residual interference between TLR4 and vaccine. Chain A and Chain B represent TLR4 and vaccine, respectively.

Molecular docking was applied to investigate the potential binding interactions between the TLR4 and the vaccination design. TLRs are expressed on the surface of a cell as a pattern recognition receptor (PRR) and identified as a specific molecular pattern of the pathogen. The Cluspro docking server was utilized to perform molecular docking to investigate the interaction between the vaccine and TLR molecules, and to determine the mode of action and efficacy of the vaccine. The server generated 10 complex models which were visualized and inspected by PyMOL v3.0 software. Complex model six (6) was chosen for further evaluation, as shown in Figure 5A. Additionally, the PDBsum server was utilized for the graphical illustration of the interference between TLR4 and vaccine molecule residues. Chain A represents TLR4 while Chain B represents the vaccine construct in a graphical image created by PDBsum. There were 24 hydrogen bonds interactions and one salt bridge, and 244 non-bonding contacts observed in TLR4-complex system, as shown in Figure 5B. The details of interaction patterns of hydrogen bond and salt bridges with binding score are given in Table 5A and Table 5B, respectively.

### 3.8. Optimization of Codon and Cloning

**Figure 6:**
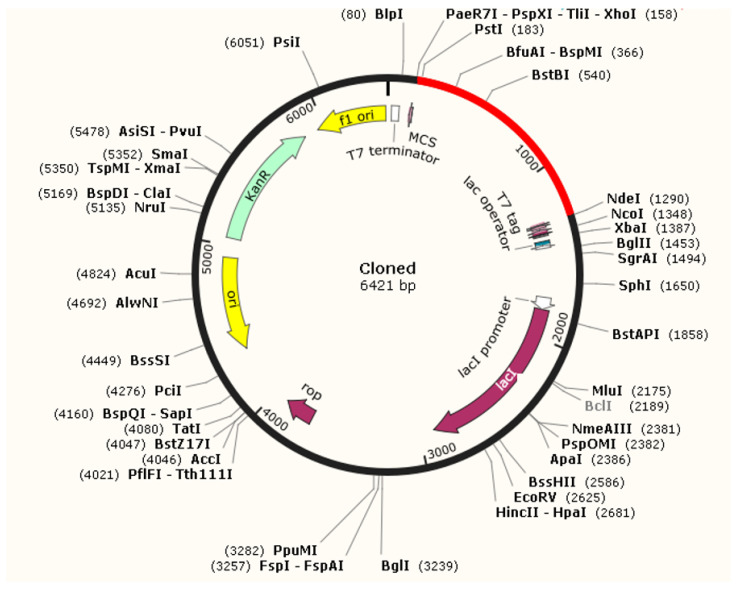
Representation of vaccine nucleotide sequence in cloning vector. Nucleotide sequence is represented in red, while vector along with different restriction site is represented in black color.

The process of codon optimization for the vaccine molecule was conducted using Jcat software, resulting in the acquisition of a 1125-nucleotide sequence for the multiepitope vaccine construct. The K12 strain of *Escherichia coli* (*E. coli*) was selected as the target organism for study in the Jcat program. The Codon Adaptation Index (CAI) and GC levels were computed and found to be 0.99 and 53.4%, respectively. Moreover, before proceeding with any additional experimental techniques, it is crucial to perform in silico cloning. The Xho1 and Nde1 restriction sites were included into the nucleotide sequence of the vaccine construct. This modified sequence was then inserted into the PET28+ vector employing SnapeGene software, as seen in Figure 6. The results showed that our gene of interst was expressed successfully.

### 3.9. Immune Simulation

**Figure 7:**
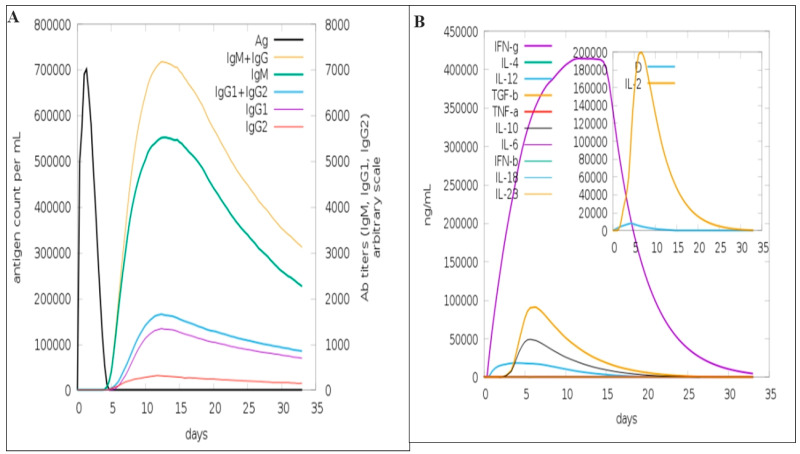
Graphical representation of immune response against OHFV vaccine. Figure (**A**) shows antibody production at different stages, while (**B**) represents production of interleukin and cytokine.

Immune simulation is a computer-based method that presents and predicts the activity of immunity and utilizes computational and mathematical models to determine the interaction between several components of immunity like antigens, antibodies, cytokines, interleukins, T cells, etc. These interactions allow the researchers to predict outcomes of a lot of immune mediated response to a particular pathogen or to determine the interaction of vaccine with immune system [20,47]. The data shown in Figure 7A revealed the induction of humoral response. It illustrated a weak response to the primary antibody and subsequently the first-time exposure, but in the second exposure, a strong IgM + IgG was observed. IgM also seems to induce a high immunological response at these stages. IgG1 played the main role in IgG response while IgG2 acted as reasonable contribution (Figure 7A). The production IFN-g actively started after the exposure of vaccine and reached the highest value and then declined after day 15. Similarly, IL-2 is shortly elevated after a few days of exposure, as shown in Figure 7B.

### 3.10. Molecular Dynamic Simulations

A 100 ns MD simulation was used to examine the time-dependent behavior of a developed multiepitope peptide vaccine construct with the TLR4 receptor in order to search for structural alterations that both interacting molecules adopted during the simulation. It was crucial to ascertain whether these alterations are significant enough to impact the construct’s docked side binding and whether the exposed epitope sequences would be recognized by the host’s immune system and trigger host immune responses. For the system that was being studied, four statistical parameters were calculated.

**Figure 8:**
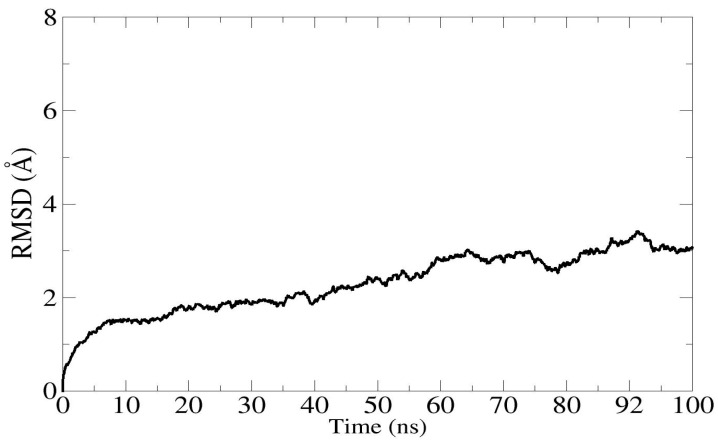
Graphical representation of root means square deviation for multiepitope vaccine construct in complex with TLR4 human immune receptor with 100 ns time intervals.

**Figure 9:**
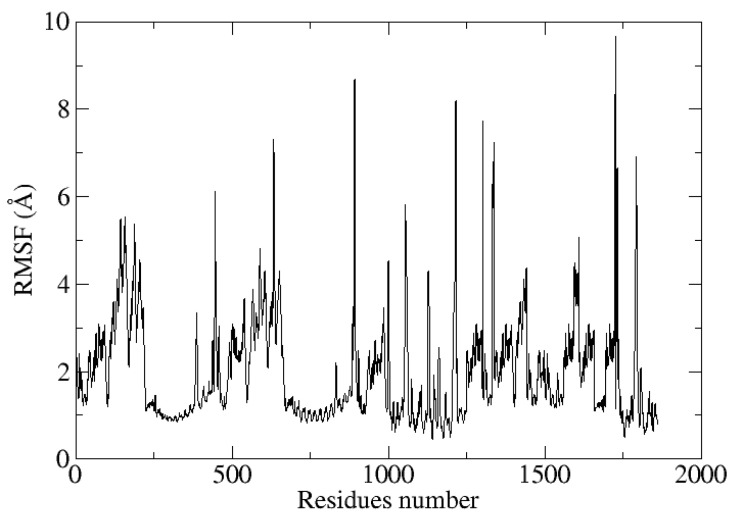
Depicting the root mean square fluctuation of the multiepitope vaccine construct with 100 ns time intervals.

Among them are RMSF and RMSD, as shown in Figure 8 and Figure 9. In order to visualize variations represented by abruptly high RMSD values at various nanoseconds, simulation trajectories were examined. Furthermore, it was demonstrated that the multiepitope peptide construct is not buried during simulation, which stabilizes the vaccine design and allows the multiepitope peptide to be exposed. The average distance between the backbone atoms of proteins that are stacked is measured by RMSD [54]. The RMSD graph shows that the complex stabilizes until 23 ns after first increasing to 3 Å at 9 ns. A further fluctuation to 4 Å in the RMSD was seen between 24 and 31 ns. With an average RMSD of 2.8 Å, the overall system shape was found to be stable (Figure 8). To obtain a deeper comprehension of amino acid flexibility, the root mean square fluctuations (RMSF) plot was assessed during the simulation. The RMSF figure showed that some amino acids in the docked complex are less flexible than others, which may be related to the vaccine’s interactions with TLR4. The vaccine displaying the TLR4 receptor’s residual stability had an average RMSF of 1.96 Å (Figure 9).

## 4. Discussion

Vaccination has a significance role in stimulation of body immune system and protects the body from disease-causing pathogens. Globally, traditional methods are employed for designing vaccine against several infectious and are considered as an effective strategy to control various diseases [55]. These traditional vaccines faces numerous challenges in contrast to in silico vaccines, which are highly stable, easy to designed, and non-toxic in nature [27,33]. Multiepitope subunit vaccines are created by carefully choosing the most immunological T cell and B cell epitopes from various proteins of a certain pathogen. These vaccines provide significant advantages over traditional vaccinations, particularly in terms of their in silico construction. It makes target-specific responses and avoids harmful non-epitopes responses. A conventional vaccine takes a significant amount of time, money, and effort to obtain the antibody-mediated response, whereas the engineering of in silico vaccine epitopes increases the binding affinity and target-specific immunogenic responses [56]. Vaccines designed through modern reverse vaccinology techniques overcome the constraints of conventional vaccines by being developed in a convenient and effective way by utilizing the genomic information of a pathogen without cultivation [27,57].

In the current study, a multiepitope vaccine against OHFV was designed on the basis of T and B cell epitope selection. Upon the recognition of the appropriate epitopes, it triggers the generation of T and B cells along with effector cells, enhancing the immunological reactions of a host upon exposure to a pathogen. If a pathogen attacks the host body, it is very crucial to activate the immune system against it. The identification of B and T epitopes is very important to the design of vaccines because they play a significant role in the activation of host immunity against viral infections [58,59,60].

In the present study, the polyprotein of OHFV was targeted for vaccine designing, which comprised both structural and non-structural proteins. Eight (8) CTL, 5 HTL, and 6 B Cell epitopes were shortlisted using different parameters like antigenicity, allergenicity, and toxicity. The details of the selected epitopes are shown in Table 1, Table 2, and Table 3, respectively.

The shortlisted epitopes were utilized to construct a multiepitope-based vaccine against OHFV. Different linkers including AAY, GPGPG, and KK were used to join the multiepitope in order to enhance the presentation and differentiation of epitopes and prevent junctional or neoepitope formation [43]. Furthermore, the human beta defensin adjuvant sequence was also added to the vaccine through the EAAAK linker at the N-terminus to improve the efficiency of the vaccine. The stability and effectiveness of the vaccine was determined by checking the physiochemical properties, allergenicity, and antigenicity. The designed vaccine was probably antigenic and non-allergenic, with score values of 0.73 and −0.93. The molecular weight of our designed vaccine is 40.6 KDa and lies in the range of an ideal vaccine, which is a molecular weight ranging from 30 to 60 KDa. The theorical PI and instability index of the designed vaccine was 9.56 and 35.27, respectively, demonstrating that our vaccine is slightly basic and stable. The Aliphatic index (AI) was 65.12, demonstrating that the vaccine is thermally stable. Furthermore, the Gand average of hydropathicity (GRAVY) value of −0.39 indicates the hydrophilic nature of vaccine [27].

The vaccine construct, after validation through different server, was the submitted to the PSIPREDV3.3 server to predict the secondary structure. A total of 16.8% alpha helix, 24.8% beta strand, and 58.4% extended coils were observed in the designed vaccine. Additionally, the tertiary (3D) structure of the vaccine was identified by utilizing the Robetta server and validated using ERRAT, PROCHECK, and ProSA-web and Pro-Q server. These servers identified errors in the vaccine’s 3D structure. The Ramachandran plot was evaluated using the ProCheck server and determined the occurrence of amino acid in the most favorable regions, which represent the reliability of 3D structure. The interference between TLR4 and the designed vaccine molecule was evaluated by the Cluspro server. TLR4 plays a crucial role in the identification of viral components, including envelope proteins, particularly in the OHFV, which is known to cause bleeding and vascular injury. This receptor can be activated to produce strong pro-inflammatory responses, which are necessary for controlling viral infections [61].

There were 24 hydrogen bonds, one salt, and 244 non-bonding contacts found in the TLR4–vaccine complex. The detail of the hydrogen bonds and salt bridges along with the binding score are given in Table 5A,B and Figure 5A,B. Immune simulation along with reverse vaccinology and immunoinformatic approaches allow better understanding about the immune response against the designed vaccine. It was determined by the server that the constructed vaccine produces large amounts of M (IgM) and Immunoglobulin G (IgG). The production of Immunoglobulin G1 and G2 (IgG1+ IgG2), Immunoglobulin M (IgM), and Immunoglobulin G1 (IgG1) occurs at the primary and secondary stages. The production of these Immunoglobulins synchronized with the reduction in antigen. The production of interleukin and cytokine was also determined. Our results indicated that the current vaccine has the capability to stimulate a high level of cytokines and humoral immune responses.

Codon optimization for the vaccine protein was performed by using Jcat (version 1.0) software to attain the elevated expression level in the K12 *E. coli* strain and increase the efficacy of transcription and translation. The obtained nucleotide sequence of the vaccine was then cloned in plasmid pET28a +, K12 strain of *E. coli* using Snapgene (version 1.1.3). To ensure the stability of the vaccine, three additional disulfide bonds were added to extremely unstable residue pairs in the improved model via the application of disulfide engineering. The vaccine was found to have a strong affinity for the TLR4 receptor, and the stability of the vaccine–TLR4 complex was further verified by MD simulations.

Prior to testing the vaccine on humans, we primarily tested animal models because of the complexity of human immunity and humans may not respond in the same way as experimental animals. The present study provides a road map for the experimental analysis and development of a potent vaccine for the prevention of OHFV using the current designed vaccine, which is more stable, safe, and highly immunogenic. Furthermore, evaluating the effectiveness of the formulated vaccine against OHFV, researchers will find this in silico architecture valuable in assessing the immunological protective effectiveness of vaccines in in vivo animal models.

## 5. Conclusions

In the present study, different immunoinformatic tools and approaches were employed to design an effective, stable, and safe vaccine construct against OHFV. The vaccine was designed in a very systemic way in which the polyprotein of OHFV was retrieved and different bioinformatic tools were used for the selection of suitable epitopes. B cell and T cell epitopes were predicted using online servers. CTL, HTL, and linear B-lymphocyte epitopes were linked with each other through different linkers. An adjuvant human beta defensin-2 was also linked through an EAAAK linker. The allergenicity, antigenicity, and physiochemical properties of the constructed vaccine were predicted and re-validated for the proposed vaccine construct. The constructed vaccine was subjected to molecular docking to determine the interaction between the vaccine molecule and Toll-like receptor-4 (TLR-4). Moreover, immune simulation was carried out to determine the interaction between the vaccine molecule and the immune system. Finally, codon optimization was carried out and the nucleotide sequence of the vaccine molecule was cloned in a pET28a+ plasmid of *E. coli*, which ensures the durability and expression of the vaccine. Furthermore, experimental validation is needed for the safety and effectiveness of the constructed vaccine against OHFV. This multiepitope peptide exhibited great affinity for the host immunoreceptor and persistent binding at the TLR4 docked side, therefore delivering epitopes to the host immune response and ensuring the triggering of innate and lasting adaptive responses. Therefore, we recommend that vaccine experts adopt the use of this this predicted vaccine construct in animal models for its biological potency to manage virus infections.

## Figures and Tables

**Table 1:** Detail of CTL epitopes with combined score, antigenicity, and toxicity score.

S. No.	Epitopes	Residue No.	MHC Binding Affinity	Rescale Binding Affinity	C-Terminal Cleavage Affinity	Transport Affinity	Combined Score	MHC-I Binding	Antigenicity	Toxicity (<0) (Non-Toxic)
1	KLKMKGLTY	576	0.1418	0.6022	0.9640	2.8820	0.8909	Yes	1.45	−0.77
2	VTDLRNCSW	994	0.1987	0.8437	0.9180	0.7970	1.0212	Yes	1.37	−0.73
3	GTDCWYAME	1103	0.2409	1.0229	0.0282	−1.9320	0.9305	Yes	1.16	−0.50
4	WTSKGTITV	1677	0.2342	0.9944	0.9409	0.2320	1.1471	Yes	1.26	−0.95
5	WSEWTNIDI	2286	0.1964	0.8337	0.5968	0.4510	0.9458	Yes	1.61	−1.21
6	VTSLGWNLI	2627	0.1649	0.7002	0.8243	0.6070	0.8542	Yes	1.33	−1.32
7	FLEFEALGF	2995	0.1492	0.6333	0.3797	2.4470	0.8126	Yes	1.77	−0.94
8	GVEGISLNY	3018	0.3920	1.6642	0.9715	2.8940	1.9546	Yes	1.40	−1.21

**Table 2:** HTL epitopes selected on the base of the properties.

S. No.	HLA	Epitopes	Percentile Rank	Antigenicity	Allergenicity	Toxicity (<0) (Non-Toxic)
1	HLA-DRB5*01:01	MEILWHAMVGTARSP	0.68	0.49	−1.74	−1.21
2	HLA-DRB3*01:01	HRDWFNDLALPWKHE	0.65	0.86	−0.44	−0.59
3	HLA-DRB1*15:01	AERLVEFGVPHAVKM	0.74	0.48	−0.66	−1.49
4	HLA-DRB1*07:01	VLHTMWHVTRGAAIF	0.89	0.53	−0.56	−0.95
5	HLA-DRB3*01:01	GELVLDTGRRIGAIP	0.59	0.63	−0.79	−1.32

**Table 3:** The B cell epitopes are evaluated based on their binding score, antigenicity, and allergenicity.

S. No.	B Cell Epitopes Sequences	Binding Score	Allergenicity	Antigenicity	Toxicity (<0) (Non-Toxic)
1	AARCPAMGPATLDEEHQSGT	0.96	−1.32	0.85	−0.65
2	KISWKSWGQSMIWSVPEAPR	0.924	−0.61	0.48	−1.32
3	SPGLLWGHRQVGVGFGSKGV	0.963	−0.44	1.48	−1.29
4	SGKRVRFHSPAVGDQQTGNA	0.771	−0.76	0.75	−0.71
5	MTATPPGKSEPFPESNGAIT	0.999	−0.48	0.77	−1.19
6	VMCDIGESSPDAAIEGERTR	0.962	−0.93	0.63	−0.49

**Table 4:** Physicochemical properties assessment of multiepitope vaccine.

S. No.	Features	Assessment	Remarks
1	Amino acids No.	375	Suitable
2	Chemical formula	C_1821_H_2817_N_517_O_508_S_19_	-
3	Molecular weight	40.6 KDa	Average
4	Theoretical PI	9.56	Slightly basic
7	Instability Index (II)	35.27	Stable
8	Aliphatic index (AI)	65.12	Thermostable
9	GRAVY (Grand Average-of hydropathicity)	−0.394	Hydrophilic

**Table 5:** The table shows hydrogen bond interactions between TLR4 and vaccine molecules along with distance in Angstrom (Å).

S. No.	Chain A		Chain B		Distance
	Residues Name	Residues Number	Residues Name	Residues Number	
1	GLU	42	ARG	12	2.84
2	GLU	42	ARG	12	2.69
3	GLU	42	CYS	11	2.92
4	ARG	264	THR	88	2.69
5	ARG	264	THR	88	2.85
6	LYS	362	TRP	84	2.69
7	LYS	402	SER	140	2.67
8	ASN	409	TYR	59	2.74
9	GLN	423	ASN	142	2.74
10	GLU	425	SER	140	2.96
11	GLU	425	ASN	142	3.04
12	ASN	433	ARG	67	3.03
13	ASN	433	VAL	63	2.72
14	LEU	434	ARG	67	2.50
15	LYS	435	LEU	66	2.80
16	ASN	448	ASN	142	2.67
17	ASP	453	TRP	116	2.98
18	HIS	458	ARG	67	2.62
19	HIS	458	ARG	67	2.55
20	HIS	458	VAL	63	2.94
21	LYS	477	GLY	115	2.69
22	THR	499	TYR	122	3.17
23	GLN	505	THR	112	2.82
24	GLN	523	TYR	122	2.88
Salt Bridges between TLR4 and vaccine Molecules					
1	GLU	42	ARG	12	2.69

## Data Availability

All the data generated in the work is presented in the manuscript.
